# CX3CL1–CX3CR1 Axis: A New Player in Coeliac Disease Pathogenesis

**DOI:** 10.3390/nu11112551

**Published:** 2019-10-23

**Authors:** Marta Fernández-Prieto, María Jesús Fernández-Aceñero, Natalia López-Palacios, Andrés Bodas, Sergio Farrais, David Cuevas, Virginia Pascual, M. Ángeles Cerón-Nieto, Saúl Horta-Herrera, Laura Espino-Paisán, Isabel Salazar, Concepción Núñez

**Affiliations:** 1 Laboratorio de Investigación en Genética de enfermedades complejas, Hospital Clínico San Carlos, Instituto de Investigación Sanitaria del Hospital Clínico San Carlos (IdISSC), 28040 Madrid, Spain; martafp87@gmail.com (M.F.-P.); david94.cg@gmail.com (D.C.); virginia.pascual.01@gmail.com (V.P.); hortaherrera.saul@gmail.com (S.H.-H.); lauraep80@gmail.com (L.E.-P.); 2 Servicio de Anatomía Patológica, Hospital Clínico San Carlos, Instituto de Investigación Sanitaria del Hospital Clínico San Carlos (IdISSC), 28040 Madrid, Spain; mariajesus.fernandez@salud.madrid.org (M.J.F.-A.); nines.ceron@gmail.com (M.Á.C.-N.); 3 Servicio de Aparato Digestivo, Hospital Clínico San Carlos, Instituto de Investigación Sanitaria del Hospital Clínico San Carlos (IdISSC), 28040 Madrid, Spain; natalia.lopa@gmail.com; 4 Servicio de Pediatría, Hospital Clínico San Carlos, Instituto de Investigación Sanitaria del Hospital Clínico San Carlos (IdISSC), 28040 Madrid, Spain; andresbpinedo@yahoo.es; 5 Servicio de Aparato Digestivo, Hospital Universitario Fundación Jiménez Díaz, 28040 Madrid, Spain; sfarraisv@quironsalud.es; 6 Departamento de Producción Animal, Facultad de Veterinaria, Universidad Complutense de Madrid, 28040 Madrid, Spain; isalazar@ucm.es

**Keywords:** coeliac disease, fractalkine, CX3CL1, CX3CR1, chemokines

## Abstract

Background: The CX3CL1–CX3CR1 axis has been related to numerous diseases. The aim of our study was to investigate its involvement in coeliac disease (CD) pathogenesis, particularly in the early phase of the disease. Methods: We collected peripheral blood from CD patients and controls, enrolled in a 3-day gluten challenge, to study soluble CX3CL1, I-TAC and MIG by Luminex, *CX3CL1* and *CX3CR1* gene expression by qPCR, and CX3CR1 protein expression in monocytes and CD8^+^, CD4^+^ and γδ^+^ T cells, by flow cytometry. We also analysed the expression of the *CX3CL1* and *CX3CR1* mRNA and protein in the duodenal biopsies of CD patients with active and treated disease, and in non-CD control individuals, by qPCR and immunohistochemistry. Results: After the gluten challenge, increased levels of CX3CL1, I-TAC and MIG proteins were observed in the peripheral blood of CD patients, with no changes in *CX3CL1* mRNA, or *CX3CR1* mRNA and protein. Regarding duodenal tissue, CX3CL1 was absent or barely present in the superficial and basal epithelium of CD patients, contrasting with the moderate to high levels present in controls. Conclusions: CX3CL1 seems to be involved in the appearance and progression of CD, and it appears to be a potential diagnostic biomarker. Its use as an alternative therapeutic target in CD deserves further research.

## 1. Introduction

Chemokines have been widely related to autoimmunity and inflammation, due to their key role in the selective recruitment of leukocytes. CX3CL1, also named *fractalkine*, constitutes a particular chemokine, which is the only member of a family that presents three amino acids between the first two cysteine residues in its primary structure. It is also notable that its mature form appears as a transmembrane protein that can be cleaved, resulting in a soluble form. Therefore, CX3CL1 can be found as a membrane-attached protein acting as an adhesion molecule, or as a soluble protein with chemotactic properties [1,2]. The soluble form can be constitutively released, but certain stimuli can accelerate the cleavage rate, which acts as a mechanism to regulate CX3CL1 soluble levels [3]. CX3CL1 exerts its function through binding to a single receptor, CX3CR1.

The CX3CL1–CX3CR1 axis has been related to numerous diseases, including several autoimmune disorders [3,4,5,6,7]. It has even been proposed as a potential biomarker and therapeutic target [8,9,10,11,12]. Some authors have related these proteins to coeliac disease (CD) [13,14,15]. CD is an immune-related systemic disease, characterized by the enteropathy triggered by gluten ingestion in genetically susceptible individuals. Overexpression of *CX3CR1* was found in the duodenal tissue and peripheral blood mononuclear cells (PBMCs) of CD patients following a gluten free diet (GFD) [13,15]. In cases of active disease, Pietz et al. [14] described increased *CX3CL1* mRNA in enterocytes, compared to controls. Additionally, they performed in vitro studies showing that CX3CL1 was induced after IFN-γ stimulation, which also caused CXCL10 (IP-10) and CXCL11 (I-TAC) increase. These last two chemokines had been previously related to CD [16,17], and it is noteworthy that they share the same receptor, CXCR3, also used by CXCL9 (MIG). CXCR3 has been suggested to be involved in the gluten-driven recruitment of immune cells to the intestinal mucosa, an important hallmark of CD [16].

IFN-γ is a key player in CD. It is involved in the initial increase and the subsequent propagation of gut permeability [18]. Most importantly, CD is a T cell mediated disorder, focusing on gluten-specific CD4 T cells, which are an important source of IFN-γ upon activation [17]. This cytokine is considered a prominent mediator of the inflammatory cascade present in CD. In fact, IFN-γ ELISPOT is used as an indirect measure of the expansion of gluten-reactive T cells after a 3-day oral gluten challenge, having been proposed as a diagnostic marker for CD [19,20]. In a previous work, we demonstrated an increase of IFN-γ and IP-10 in the peripheral blood of CD patients six days after starting such a gluten challenge protocol. In the present study, we aim to investigate the role of the CX3CL1–CX3CR1 axis in CD, as well as its potential involvement, together with CXCR3 ligands, as diagnostic markers in the first steps after a gluten challenge.

## 2. Materials and Methods

### 2.1. Subjects

Five groups of individuals were considered: CD patients with active (aCD), or treated (tCD), disease; disease controls with normal histology or with histological lesions (Marsh 3); and healthy controls. The number and characteristics of individuals in the different categories are specified for each analysis and shown in Table 1.

Both paediatric and adult patients were included. CD diagnosis was performed according to the current guidelines at every moment, based on clinical presentation, serological and HLA genetic markers, and histological examination [21,22]. Treated disease was confirmed by negative anti-TG2 serology and histological recovery. Healthy controls correspond to healthy volunteers with negative anti-TG2 antibodies, who followed a GFD for at least one month to participate in the study. Disease controls correspond to individuals being studied for anaemia or suspected CD, who showed normal mucosa in the duodenal biopsy; those with evaluated CD serology (most cases) showed negative results. Regarding the disease controls with atrophy analysed by immunohistochemistry, two showed an absence of EMA antibodies, non-HLA-DQ2.5/DQ8 genetics and no response to the GFD; the third showed negative antibodies and spontaneous mucosal recovery, having followed a diet containing gluten.

### 2.2. Gluten Challenge

Some participants were exposed to three days of gluten, according to previous publications, [23,24] following a protocol previously described [24]. Briefly, after at least one month on a GFD, individuals consumed approximately 10–14 g of gluten every day for three consecutive days. Blood was collected before starting gluten intake (day 0) and after six days (day 6).

### 2.3. Cytokine Analysis

Serum samples were kept frozen until used to measure CX3CL1, I-TAC and MIG. These proteins were measured in four CD patients and 11 healthy controls, before and after six days of a 3-day gluten challenge, in a Luminex assay, using Bio-Plex MAGPIX (Bio-Rad, Hercules, CA).

### 2.4. Gene Expression

mRNA was extracted from the peripheral blood of 10 tCD patients and five healthy controls enrolled in the 3-day gluten challenge. However, for technical reasons, paired samples at day 0 and 6 of the challenge were only available from five patients and three controls, and for the remaining patients we only had data from one day. mRNA from four CD patients following a gluten-containing diet was also obtained. In addition, the duodenal tissue of eight CD patients (five with aCD and three with tCD) and nine disease controls was used for gene expression analysis.

Peripheral blood was collected in PAXgene blood RNA tubes (Qiagen, Westburg, The Netherlands) and kept frozen at -20 °C until use. RNA was obtained using the NucleoSpin RNA Blood kit (Macherey–Nagel GmbH & Co. KG, Dueren, Germany). Tissue samples were immersed in RNA later, after being obtained under routine endoscopy for diagnosis or follow-up. These collected samples were kept at 4 °C for 24–48 hours, and subsequently stored at −80 °C until use. RNA was obtained using the RNeasy Mini Kit (Qiagen). In both cases, cDNA was obtained by RT–PCR reaction (High Capacity RNA-to-cDNA Master Mix, Applied Biosystems, Foster City, CA, USA).

The mRNA expression of *CX3CL1* and *CX3CR1* was measured by quantitative PCR (qPCR) using TaqMan assays Hs00171086_m1 and Hs01922583_s1, respectively, and Hs99999902_m1 (*RPLP0)* and Hs99999908_m1 (*GUSB*) (Applied Biosystems, Foster City, CA), as endogenous reference genes for intestinal and blood expression, respectively. Expression levels were calculated according to delta–Ct (ΔCt = Ct target−Ct endogenous).

### 2.5. Immunohistochemistry

We collected data from seven paired samples of the same patients with active (Marsh 3) and treated (Marsh 0) CD, and 13 disease controls, 10 with normal mucosa (Marsh 0) and three showing intestinal atrophy (1 Marsh 3a, 1 Marsh 3b and 1 Marsh 3c).

Antigen retrieval for immunohistochemistry was performed in PT–Link (Dako, Glostrup, Denmark) for 20 minutes at 95 °C, in a low-pH buffered solution (Dako). Endogenous peroxidase was blocked by immersing the sections in 0.03% hydrogen peroxide for 5 minutes. Slides were washed for 5 minutes with a Tris buffered saline solution, containing Tween 20 at pH 7.6 and incubated with the primary antibodies anti-CX3CL1 (1/75; Abcam, Cambridge, UK) and anti-CX3CR1 (1/75; Abcam) overnight at room temperature, followed by incubation with the appropriate anti-Ig horseradish peroxidase-conjugated polymer (EnVision, Dako, Denmark) to detect antigen–antibodies. Sections were then visualized with 3,3’-diaminobenzidine as a chromogen for 5 minutes and counter-stained with hematoxylin. Both antigens were expressed in the cytoplasm of the epithelial cells, the fibroblasts in the lamina propria, and in inflammatory cells, and expression was scored independently by two pathologists blinded to the clinical diagnosis of the patients. In all cases, the intensity of the immunohistochemical expression was graded as no stain, weak, moderate or intense (0, 1, 2 or 3, respectively).

### 2.6. Flow Cytometry

CX3CR1 was analysed in 14 CD patients and 14 controls (four healthy controls and 10 disease controls) following a GFD and participating in the 3-day gluten challenge study.

A volume of 250 μ: of whole peripheral blood was labelled with 3 μL of each of the following monoclonal antibodies: PE/Cy7 anti-human CX3CR1 (clone 2A9-1), BV421 anti-human TCRγδ (clone B1), APC anti-human CD8 (clone SK1) and APC Cy7 anti-human CD3 (clone HIT3a), all from BioLegend (San Diego, CA). Samples were incubated at 4 °C in the dark for 20 minutes, and mixed with 3 mL of FACs Lysing BD Biosciences reagent to lyse the erythrocytes. Flow cytometry acquisition was performed on a FACSCanto II flow cytometer (BD Biosciences, Franklin Lake, NJ) and analysis was done using the flow cytometry software Infinicyt 2.0 (Cytognos, Salamanca, Spain). When the number of CD8^+^T cells was below 50,000, 250 additional μL of blood were labelled and appended to the previously acquired data.

Selection of monocyte and lymphocyte populations was based on their forward and side scatter properties. Most of them showed the CX3CR1 receptor, but two differentiated CX3CR1^+^ populations could be observed, which we classified as CX3CR1^low^ and CX3CR1^hi^ populations. In addition, a small but variable CX3CR1^−^ population was present. Regarding lymphocytes, only CD3^+^ cells (T cells) were analysed. Among them, CD8^+^ or γδ^+^ cells were selected based on the presence of these markers, and CD3^+^CD8^−^γδ^−^ cells were assigned as CD4^+^ lymphocytes [25].

### 2.7. Statistical Analysis

Data of soluble CX3CL1, I-TAC and MIG in sera were analysed as previously described [24]. Briefly, a MIXED procedure in a 2 × 2 factorial design was used in the SAS software, with factors being the category group (CD and controls) as a fixed factor, the sampling time (day 0 and day 6) as a repeated measure, and the interaction between both of them. To keep the significance level at 0.05, despite performing multiple comparisons, significant p values were considered below 0.013.

Flow cytometry data were analysed with a similar procedure. In a 2 × 4 factorial design, we considered the category group as a fixed factor, the sampling time as a repeated factor, and their interaction. Individual analyses were performed for CX3CR1^−^, CX3CR1^low^ and CX3CR1^hi^ populations from each cell type, considering two different variables: cell count and mean fluorescence intensity (MFI). MFI was also analysed through the MIXED procedure of SAS, while cell counts followed a binomial distribution, and the GLIMMIX procedure implemented in SAS was used. To keep the significance level at 0.05, despite performing the multiple comparisons necessary to analyse cell count, significant p values were considered below 0.0085.

Gene expression and immunohistochemistry data were analysed using a Student’s *t* test to compare mean values when normality was confirmed. When this distribution could not be assumed, non-parametric tests were used—the Wilcoxon test or the Mann–Whitney *U* test—for paired and independent samples, respectively. Normality was assessed with the Kolmogorov–Smirnov test. SPSS v15 was used for calculations.

Correlation between parametric quantitative variables was calculated with the bivariate Pearson correlation coefficient, using the SPSS software.

### 2.8. Ethical Statement

Written informed consent was obtained from all participants or their legal guardian before starting the study. The Ethical Committees of the participant hospitals approved the study.

## 3. Results

### 3.1. Cytokine Analysis

A significant *p* value for the interaction between groups (four tCD and 11 controls) and time of sampling (day 0 and 6 of the gluten challenge) was obtained for the three soluble chemokines analysed (Table 2).

Further analysis showed significantly increased levels in the sera of CD patients after the gluten challenge. When comparing CD patients and controls, no differences were observed at day 0, but in all cases a significant difference was observed at day 6 between both groups, at α = 0.05, although only I-TAC remained significant after adjustment for multiple comparisons.

**Figure 1:**
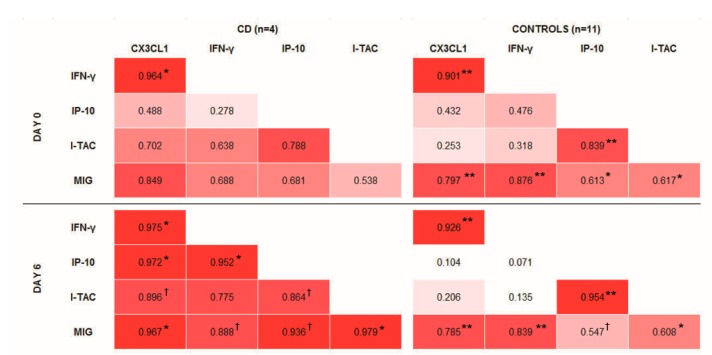
Bivariate correlations between the soluble markers studied. Pearson´s coefficient has been used to establish a colour scale, because *p*-values vary depending on the sample size. ^†^ *p* ≤ 0.1; * *p* < 0.05; ** *p* < 0.01.

Bivariate correlations in controls were not changed after the gluten challenge, and resemble those present in tCD, i.e., at day 0 of sampling (Figure 1). CX3CL1 was positively correlated with IFN-γ and MIG, and a positive correlation was also observed between IP-10 and I-TAC. After the gluten challenge, it was notable that all the studied cytokines became positively correlated in the group of CD patients.

### 3.2. Gene Expression

In blood, *CX3CR1* showed an expression of around 1000 times higher than *CX3CL1.* In duodenal tissue, *CX3CL1* was predominant, with an expression of around 10 times higher than its cognate receptor.

**Figure 2:**
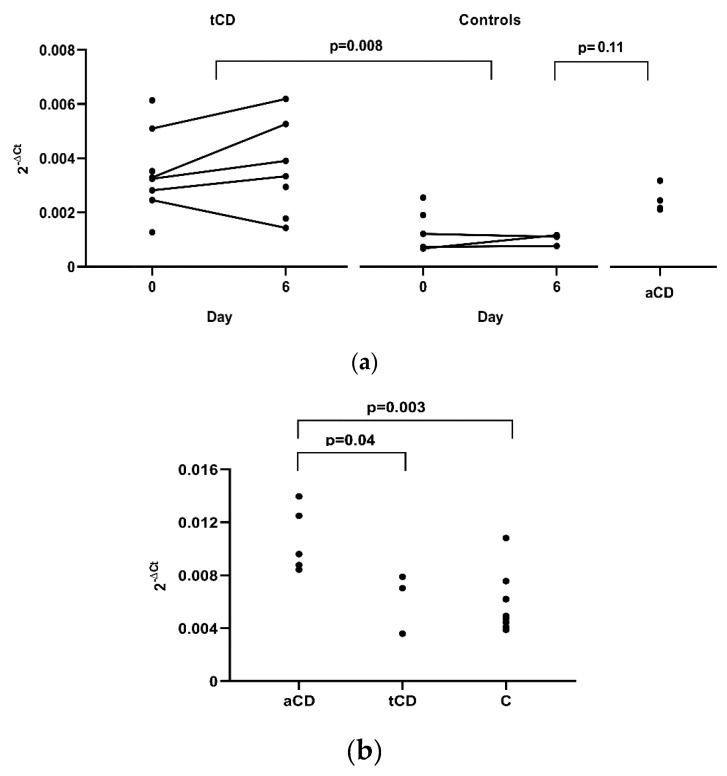
Gene expression patterns of *CX3CL1*. Data correspond to 2^−^^Δ^^Ct^, i.e., the relative expression of *CX3CL1* mRNA compared with the endogenous gene in (**a**) peripheral blood of patients with coeliac disease (tCD) and healthy controls, following a gluten free diet and enrolled in a 3-day gluten challenge; and CD patients following a gluten containing diet (aCD); (**b**) duodenal tissue of patients with active (aCD) or treated (tCD) CD, and disease controls (C).

Differences before and after the gluten challenge for *CX3CL1* and *CX3CR1* in peripheral blood were not observed in tCD patients (five samples) or in controls (three samples). However, a significant difference in results was obtained when comparing *CX3CL1* expression between these two groups, with a higher expression in tCD samples where data from only day 0 (three CD patients and two controls), or day 6 (two CD patients), were used, and the mean of day 0 and 6, when both measures were available (five tCD patients and three controls) (*p* = 0.008, Figure 2a). We also analysed four CD patients following a gluten containing diet and we again observed higher *CX3CL1* gene expression in aCD, although with only a borderline significance when compared to controls (*p* = 0.11). In duodenal tissue, a higher expression of *CX3CL1* was observed in aCD when compared to tCD (*p* = 0.04) and to non-CD controls (*p* = 0.003) (Figure 2b).

No differences were observed when studying *CX3CR1* expression.

### 3.3. Immunohistochemistry

**Figure 3:**
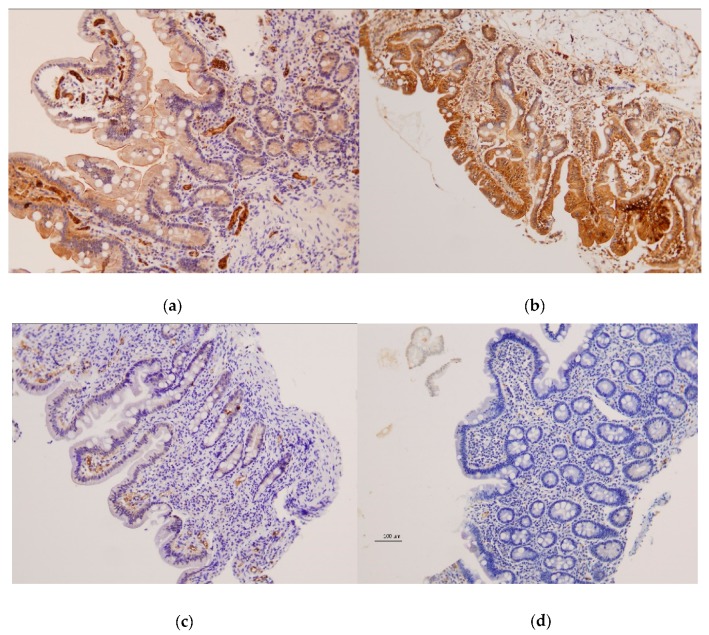
Protein expression of CX3CL1 in duodenal tissue obtained by immunohistochemistry (10×). Data correspond to paired samples of a non-coeliac disease (CD) individual with (**a**) normal histology and (**b**) atrophy; and a CD patient with (**c**) normal histology and (**d**) atrophy.

**Figure 4:**
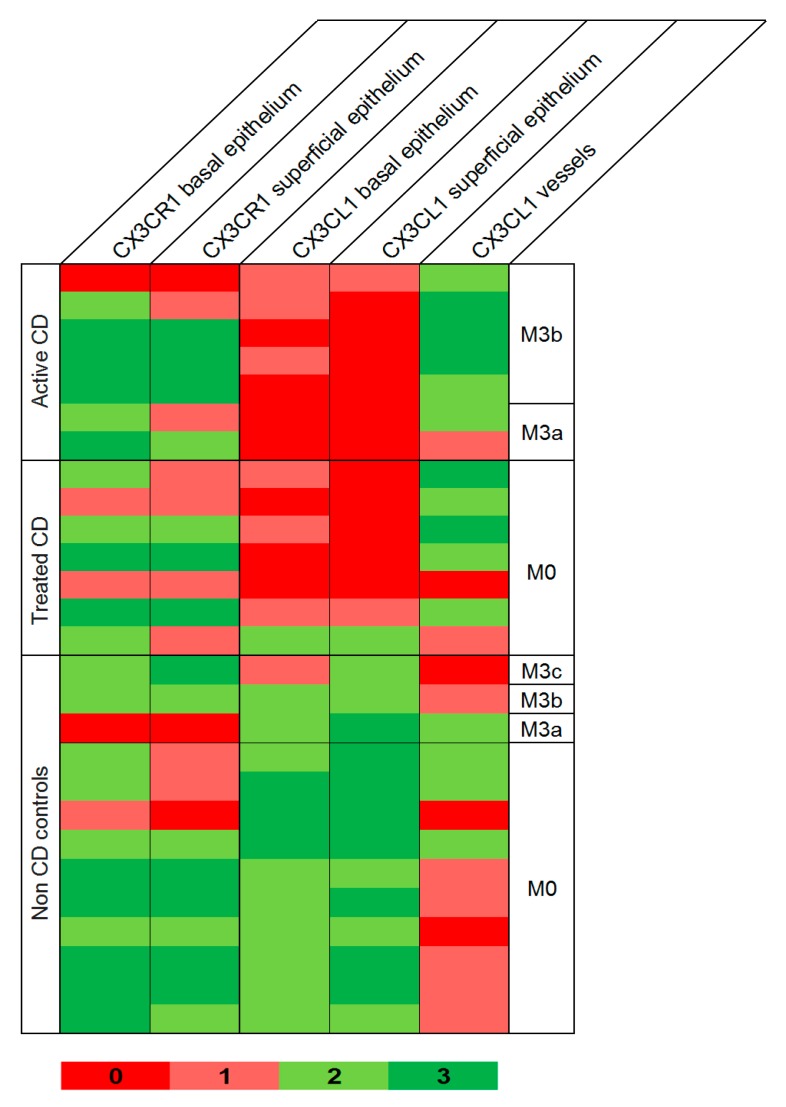
Protein expression of CX3CL1 and CX3CR1 in duodenal tissue detected by immunohistochemistry of all the patients with active and treated coeliac disease and non-coeliac controls analysed. Expression pattern: no stain, 0; weak, 1; moderate, 2; or intense, 3. CD coeliac disease, C controls, Marsh 0/3a/3b/3c, respectively.

Examination of duodenal samples showed a variable expression of CX3CR1 in superficial and basal intestinal epithelium in both patients and controls, with no differences between groups. CX3CL1 was also observed in superficial and basal epithelium, but highly significant differences were detected between CD patients and controls (Figure 3 and Figure 4).

CX3CL1 was absent or barely present in CD patients with active disease, contrasting with the moderate to high expression present in non-CD controls, either with Marsh 0: *p* < 10^−4^, or showing atrophy: *p* = 0.008 and *p* = 0.067 for superficial and basal epithelium, respectively. Subjects with tCD also showed lower CX3CL1 than controls, although some patients showed normal levels, and differences between groups were less significant: *p* = 0.0003 and *p* = 0.0004 for superficial and basal epithelium, respectively, vs. controls with Marsh 0: *p* = 0.025 and *p* = 0.22 for superficial and basal epithelium, respectively, vs. controls with atrophy. CX3CL1 was also observed in blood vessels, with a higher expression of aCD and tCD when compared to non-CD controls (*p* = 0.003 and *p* = 0.07, respectively).

### 3.4. Flow Cytometry

CX3CR1 was present in almost all cells analysed in the blood, and was only absent in less than 2% of each considered T cell subset (CD8^+^, CD4^+^ and γδ^+^), and in less than 10% of the monocytes. Data analyses did not show significant results when analysing CX3CR1 by flow cytometry, either before and after the gluten challenge, or between CD patients and controls for any of the studied cell types. 

## 4. Discussion

The expression of chemokines beyond certain stimuli, and their contribution to the inflammatory process, make them good candidates to better understand the pathogenesis of inflammatory or immune-related conditions, and to become biomarkers for disease diagnosis. Therefore, we investigated the possible role of CX3CL1 and other IFN-γ-induced chemokines in the early phase of CD, by studying their changes after a gluten challenge. IFN-γ and IP-10 increased in the peripheral blood of CD patients six days after starting a 3-day gluten challenge [20,24]. In this work, we observed the parallel increase of CX3CL1, I-TAC and MIG. A constitutive positive correlation seems to exist between IFN-γ and CX3CL1, and, at a lower intensity, between these and MIG. IP-10 and I-TAC are also positively correlated (Figure 1). Therefore, regulated levels of these cytokines are probably required to maintain homeostasis. In CD, that regulation is altered by gluten, and the molecules increase in a similar manner, becoming highly correlated. The observed higher levels of CX3CL1 do not seem to be due to increased gene expression, since no difference in levels of mRNA were observed before and after the gluten challenge. However, the induction of mRNA and CX3CL1 membrane-bound protein precede the presence of soluble CX3CL1 [26], which could preclude us from seeing mRNA changes at day 6. Interestingly, a basal higher *CX3CL1* gene expression seems to exist in CD. This may generate higher levels of the CX3CL1 membrane-attached form, which could be quickly released into the bloodstream after certain stimuli. We demonstrate that gluten-induced IFN-γ seems to be one of those stimuli. It should be noted that activated CD4, CD8 and γδ T cellswith gut-homing receptors are also observed in the blood of CD patients at day 6 of the 3-day gluten challenge [23,24,27]. Due to the chemotactic properties of the studied analytes, CX3CL1, coordinated with, at least, IP-10, I-TAC and MIG, would contribute to the recruitment of lymphocytes to the intestinal epithelium, which characterizes the early phase of CD. These last three chemokines, all sharing the same receptor, CXCR3, have been previously described as participating in cell recruitment to the inflamed tissue in CD [16]. Our work places these chemokines as potential diagnostic biomarkers that can be especially useful after a short gluten challenge.

In addition, we investigated the role of CX3CL1–CX3CR1 in the target tissue. As previously described [26], we have observed CX3CL1 expression in epithelial and endothelial cells in the small intestine. An important finding is the scarce presence of CX3CL1 in the duodenal epithelium of CD patients, contrasting with the moderate to high levels observed in individuals studied with suspected CD, showing normal mucosa. This was observed in patients with aCD and in most of the treated patients with normal mucosa (Figure 4). The histological alterations present in aCD are characteristic of, but not pathognomonic for, the disease. This generates cases of uncertain diagnosis, such as in those showing duodenal lesions in the absence of specific antibodies (seronegative CD). Further confirmation of our results in a new set of patients is needed, but our results open a door to a possible new tool to support CD diagnosis. Immunohistochemistry is widely used in clinical diagnostics, and duodenal biopsies are required when testing for CD in adults and in children with uncertain diagnosis. Therefore, analysis of CX3CL1 represents a feasible tool that would not need additional sampling. Since immunohistochemistry can be performed in paraffin-embedded tissue, this could be also used in patients still waiting for a definitive diagnosis. It could also be a valid test for the increasing number of individuals who start a self-prescribed GFD without previous CD evaluation, because CX3CL1 can remain at low or null levels even after starting treatment. 

Epithelial cells have been described as the source of soluble CX3CL1 and the other studied chemokines [14,16,26], which would create a gradient to direct lymphocyte migration to the intestinal mucosa. Interestingly, Muehlhoefer et al. [26] demonstrated that intestinal intraepithelial CX3CR1^+^ lymphocytes could be chemoattracted by soluble CX3CL1, but not by its membrane-attached form. They also observed, using the intestinal epithelial cell line T84, that most of the CX3CL1 expressed in non-stimulated T84 cells corresponded to the membrane-bound form, but after stimulation the highest increase corresponded to the soluble form. Thus, the lack of CX3CL1 in the epithelium of CD patients could be caused by its cleavage into the soluble form, which may be hard to distinguish by immunohistochemistry, and it would then act as a chemoattractant. In vessels, the observed increased CX3CL1 expression can be explained by the role of this CX3CL1-membrane form as an adhesion molecule, which arrests *CX3CR1*^+^ leukocytes from flowing blood and increases the likelihood of their crossing the endothelial barrier, thus infiltrating the target tissue.

We evidenced by flow cytometry that CX3CR1 was present in the great majority of monocytes and lymphocytes in peripheral blood, which enables their recruitment following a CX3CL1 gradient. However, in contrast with the observations regarding CX3CL1, it seems that regulation of the cognate receptor is not altered in CD. No differences were found between CD patients and controls, before and after the gluten challenge, as observed by flow cytometry and gene expression in blood. In addition, we did not observe differences at the intestinal level between CD and non-CD patients, according to immunohistochemistry and gene expression analyses. Nevertheless, down-regulation of *CX3CR1* expression has been described in CD subjects following a GFD [13,15], and this group needs further evaluation with an increased sample size. The effect on individuals with a treated disease is in accordance with our results of CX3CL1 by immunohistochemistry.

In summary, our work points toward the role of the CX3CL1–CX3CR1 axis in CD, as has been previously suggested [13,14,15]. However, some discrepancies exist with those studies. Besides those regarding *CX3CR1* gene expression, differences are also found in immunohistochemistry results, but it is notable that Pietz et al. [14] did not observe CX3CL1 expression in the small bowel of controls. One limitation of our study is the number of individuals analysed, which needs to be increased in further research. In addition, a majority of those included were adult women. Although no differences seem to exist based on age or sex, higher numbers of paediatric and male patients should be studied in future.

In the last decade, CX3CL1 has raised considerable interest, as it is the first chemokine that is able to mediate leukocyte attraction, capture and firm adhesion, in the absence of integrins. Its role recruiting inflammatory cells to the target tissue confers it especial relevance to initiating and propagating inflammation and disease. In this respect, its potential use as a biomarker and therapeutic target has been studied under numerous conditions.

## 5. Conclusions

Our work evidences a potential use of CX3CL1 for CD diagnosis and suggests the possibility of investigating its use as an alternative treatment to the GFD, owing to its role in CD pathogenesis.

## Figures and Tables

**Table 1:** Age and sex of the studied subjects.

Study	Children				Adults			
	*N*	Age (Years)		% Women	*N*	Age (Years)		% Women
		Range	Mean ± SE			Range	Mean ± SE	
Cytokine analysis								
tCD *					4	34–64	48 ± 7	50
Controls					11	27–40	34 ± 1	90
Gene expression blood								
aCD	1	1	1	0	3	21–48	30 ± 11	100
tCD *					10	21–56	39 ± 3	40
Controls					5	35–51	40 ± 3	100
Gene expression biopsy								
aCD	1	16	16	100	4	26–43	35 ± 3	75
tCD	1	15	15	0	2	48–64	56 ± 8	67
Controls	2	7–11	9 ± 2	50	7	17–69	45 ± 8	86
Immunohistochemistry								
aCD and tCD					7	33–68	53 ± 5	71
Controls Marsh 0					10	38–83	55 ± 6	60
Controls Marsh 3	1	9	9	0	2	40–62	51 ± 11	0
Flow cytometry								
tCD *					14	17–70	38 ± 4	71
Controls	2	6–12	9 ± 3	0	12	17–52	33 ± 3	92

aCD, active coeliac disease; tCD, treated coeliac disease; SE standard error; tCD * patients who participated in a 3-day gluten challenge.

**Table 2:** Comparison of soluble protein levels (mean ± SE) in sera of CD patients and controls enrolled in a 3-day gluten challenge. Non-significant values are not shown.

Chemokine	CD		Controls		*p* Interaction	*p* Day 0 vs. 6 CD	*p* CD vs. ControlsDay 6
	Day 0	Day 6	Day 0	Day 6			
CX3CL1	174.5 ± 16.7	247.1 ± 16.7	170.0 ± 16.7	167.1 ± 27.6	0.0022	0.0009	0.0276
I-TAC	41.1 ± 7.8	156.9 ± 26.7	28.5 ± 4.7	27.8 ± 16.1	0.0011	0.0003	0.0012
MIG	422.3 ± 95.9	1087.5 ± 275.4	333.7 ± 57.8	286.5 ± 166.1	0.0165	0.0102	0.0271

Values are expressed in pg/mL. CD, coeliac disease.
